# Effects of equipment and technique on peak flow measurements

**DOI:** 10.1186/1471-2466-6-14

**Published:** 2006-06-20

**Authors:** Thomas Bongers, B Ronan O'Driscoll

**Affiliations:** 1 School of Clinical Sciences, University of Liverpool, Liverpool, UK; 2 Dept of Respiratory Medicine, Hope Hospital, Salford, UK

## Abstract

**Background:**

Different lung function equipment and different respiratory manoeuvres may produce different Peak Expiratory Flow (PEF) results. Although the PEF is the most common lung function test, there have been few studies of these effects and no previous study has evaluated both factors in a single group of patients.

**Methods:**

We studied 36 subjects (PEF range 80–570 l/min). All patients recorded PEF measurements using a short rapid expiration following maximal inspiration (PEF technique) or a forced maximal expiration to residual volume (FVC technique). Measurements were made using a Wright's peak flow meter, a turbine spirometer and a Fleisch pneumotachograph spirometer.

**Results:**

The mean PEF was 8.7% higher when the PEF technique was used (compared with FVC technique, p < 0.0001). The mean PEF recorded with the turbine spirometer was 5.5% lower than the Wright meter reading. The Fleisch spirometer result was 19.5% lower than the Wright reading. However, adjustment of the Wrights measurements from the traditional Wright's scale to the new EU Peak Flow scale produced results that were only 7.2% higher than the Fleisch pneumotachograph measurements.

**Conclusion:**

Peak flow measurements are affected by the instruction given and by the device and Peak Flow scale used. Patient management decisions should not be based on PEF measurement made on different instruments.

## Background

It is customary for spirometers to print out Peak Expiratory Flow (PEF) measurements as well as measurements of Forced Expiratory Volume in one second (FEV1) and Forced Vital Capacity (FVC). It is not known if these PEF measurements correspond to those made on Wright's or Mini-Wrights meters. Previous studies have found that different spirometers and different Peak Flow Meters can record PEF differently with error rates of up to 26% in laboratory calibration tests [1]. Furthermore, some spirometers use a traditional Wright scale to record PEF whilst others use a scale which corresponds more closely to the new European scale which is closer to the true PEF measured in laboratory studies [2].

The Wright Peak Flow Meter was developed to measure Peak Expiratory Flow [3]. This requires the subject to exhale as quickly as possible into a recording device following maximum inspiration. Maximal expiratory flow lasts for only a fraction of a second and occurs very early in expiration. It is not necessary for the subject to continue exhaling to residual volume. For most subjects, a short but forceful blow will be sufficient to register the maximal expiratory flow ("PEF technique").

The FVC measurement requires a blow that starts from maximal inspiration and proceeds to residual volume. Although PEF measurements and FEV1/FVC measurements both require a rapid exhalation, the instructions given to the subject are different and it is possible that the two techniques are not interchangeable.

Wensley and colleagues found that the PEF of children was 3% higher when measured using a PEF technique compared with a FVC technique on a turbine spirometer [4]. They concluded that this small difference was not clinically significant but they did not compare the measurement made on a Peak Flow meter. In our chest clinic, technicians record the FEV1/FVC of all patients using a Fleisch pneumotachograph-based spirometer and the Peak Flow is measured by the same technician using a Wright's Peak Flow Meter using "PEF technique". We noticed that the peak flow measurement from the two devices differed by up to 20%.

To explore these issues further, we studied the effect of the instruction given to adult subjects (PEF technique or FVC technique) when the peak expiratory flow was measured. We further investigated the effect of different devices using a Wright's Peak Flow Meter and two types of spirometer (turbine spirometer and Fleisch pneumotachograph). This allowed comparison of the instrument effect and the instruction effect using a group of subjects with a wide range of peak flow values.

## Methods

We invited 38 sequential patients attending a hospital chest clinic to participate in a study comparing two different Peak Flow techniques on three devices. Informed consent was obtained from all patients willing to participate. Exclusion criterion was inability or unwillingness to take part in the study. Two patients declined and 36 agreed to take part in the study; clinical details are given in table 1.

The devices used were as follows: standard Wright's Peak Flow Meter (Clement Clarke International, Harlow, CM20 2TT, UK), Micro-Medical Microlab 3300 turbine spirometer (Micro Medical Ltd Rochester, ME1 2AZ, UK) and Vitalograph Spirotach III (Fleisch pneumotachograph) spirometer (Vitalograph Ltd Buckingham MK18 1SW, UK).

The Micro-Medical turbine spirometer is calibrated to match a standard Wright's PEF scale and the Vitalograph pneumotachograph is calibrated to read a "true PEF" which is similar to that measured with the new EU PEF scale (personal communication Micro Medical Ltd UK and Vitalograph Ltd UK). All instruments were serviced and calibrated regularly in accordance with the manufacturers' instructions.

Each patient undertook 12 expiratory manoeuvres. This consisted of duplicate measurements of peak expiratory flow using PEF technique and FVC technique with each of the above three devices. The instruction given was standardized. For "PEF technique", the subject was instructed to inhale as deeply as possible and to blow out as fast as possible into the mouthpiece. The best of two technically satisfactory blows into each instrument (as judged by the investigator) was recorded. For "FVC technique", the subject was instructed to inhale as deeply as possible and to blow forcefully into the mouthpiece as fast as possible until their lungs were empty. The individuals were randomly allocated to one of six groups. Each group consisted of 6 participants and had a pre determined order of technique and device to be used.

Group 1: A - C - E - B - D - F

Group 2: C - E - A - D - F - B

Group 3: E - A - C - F - B - D

Group 4: B - F - D - A - E - C

Group 5: F - D - B - E - C - A

Group 6: D - B - F - C - A - E

The letters A - E represent the different devices and techniques used as follows.

A = Wright meter with Peak flow technique times two

B = Wright meter with FVC technique times two

C = Vitalograph Fleisch Spirometer with Peakflow technique times two

D = Vitalograph Fleisch meter with FVC technique times two

E = Micro-Medical Microlab 3000 Turbine Spirometer with Peak flow technique times two

F = Micro-Medical Microlab 3000 Turbine Spirometer with FVC technique times two

This ensured that no "order effect" could occur if patients became fatigued during their final blows. The best of two technically satisfactory measurements was used for each of the six manoeuvres. Recordings from the Wright's meter were entered twice, once from the scale on the meter itself (Wright's scale) and also after conversion to the new EU PEF scale. [2] Predicted PEF values were derived from the European Coal and Steel Community equations [5].

Ethics approval for the study was obtained from the Salford and Trafford Local Research Ethics Committee.

All measurements were entered into a scientific database (GraphPad Prism 3, GraphPad Software, San Diego, California CA 92130 USA) and the mean and median values for each technique on each instrument were calculated. As the results met criteria for normality, all results are expressed as means and compared by t-tests. The primary end point of the study was to determine if the PEF was different when recorded with different instruments and with different instructions to the patient.

## Results

**Figure 1:**
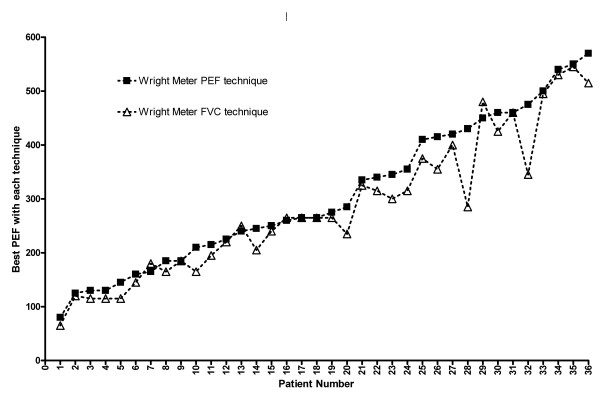
**Wright meter – both techniques**. Comparison of PEF measurements on Wright Peak Flow meter using "Peak Flow technique" (square symbols) or "FVC technique" (triangles). The values shown are the best of two measurements with each technique. Patients are arranged on the basis of increasing PEF measured using the "PEF technique".

**Figure 2:**
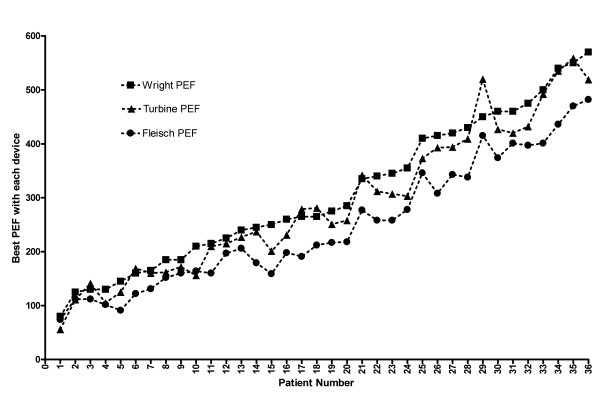
**Three devices using "peak flow technique"**. Comparison of PEF measurements using "Peak Flow technique" on three devices; Wright Peak Flow meter (square symbols), Micro-Medical turbine spirometer (triangles) and Vitalograph Fleisch pneumotachograph spirometer (circles). The values shown are the best of two measurements with each device. Patients are arranged on the basis of increasing PEF measured using the Wright's meter with "PEF technique".

36 patients completed the study; clinical details are given in table 1. Mean results for each device are shown in table 2 and in figures 1, 2.

For all devices combined, the mean of 216 PEF recordings using the Peak Flow technique was 8.7% higher than 216 recordings using the FVC technique and this difference was similar for all three devices; p < 0.0001 (table 2). The difference attributable to technique was lower with the low-resistance Fleisch device (mean 5.5% difference) compared with the higher resistance Wrights' device (mean 7.8% difference) and the Turbine device (mean 11.3% difference). For most subjects, there was little or no difference in recorded PEF between the two techniques but, for three patients, there was a substantial difference of >50 L/min (patients 26, 28 and 32 in figure 1). Removal of these three patients would result in a mean 5.3% difference in PEF for the other 33 patients. (mean result with PEF technique 296 L/min, FVC technique 280 L/min, p < 0.001).

**Figure 3:**
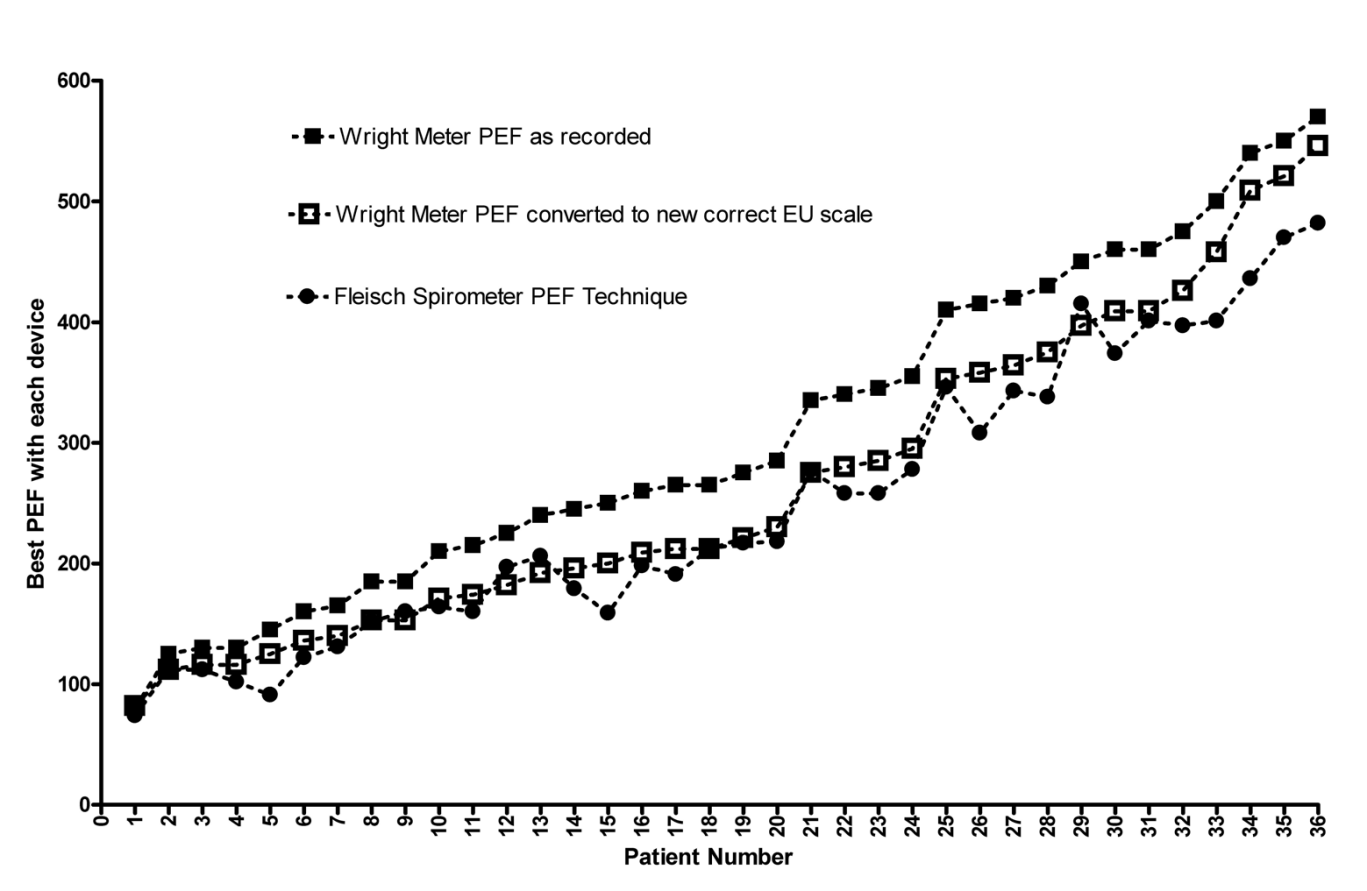
**"Peak Flow technique" with Wright meter and Fleisch pneumotachograph spirometer and conversion to new EU scale**. Comparison of PEF measurements using "Peak Flow technique" on Wright meter (closed square symbols) or Fleisch pneumotachograph spirometer (circles). The open squares are Wright values converted to the new EU scale. The values shown are the best of two measurements with each technique. Patients are arranged on the basis of increasing PEF measured using the Wright meter.

Comparing the same technique on each of three devices, the turbine spirometer gave a PEF which averaged 5.5% lower than that recorded by the Wright's meter and the Fleisch pneumotachograph device gave a reading that was, on average 19.5% lower than the Wright's meter reading (figure 2). However, the pneumotachograph measurement was much more closely matched to the "corrected Wright's" value on the new EU PEF scale (figure 3). This effect appeared to be symmetrical across a wide range of peak flow values from below 100 l/min to above 500 l/min. However, the converted PEF (EU scale) was closer to the Wright's scale than to the Fleisch pneumotachograph reading for 12 patients with Wright's PEF above 400 L/min.

## Discussion

This is not the first study to compare the results of different devices for the measurement of PEF but it is the first study to look at the combined effect of device and instruction given in a group of adult patients with a wide range of lung diseases and a wide range of PEF values. The present study confirms previous reports that the instruction given, the device chosen and the scale used on the device can all affect the PEF measurement [1,2,4,6-10].

The instruction given (PEF technique or FVC technique) had a small effect for most patients (5.3% lower with FVC technique) but there was a major effect of >50 l/min for three of 36 patients which was reproducible on 3 different devices. The overall difference in PEF (8.7%) was greater than the 3% difference reported in a previous study of children with normal peak flows [4]. This may be explained by adult patients having a better understanding of the subtle difference in the instructions given. The difference in PEF attributable to the instruction given was smallest for the low resistance Fleisch device.

The different lung function devices gave different values for PEF even when the same technique was used with each device. This difference (up to 19%) was in line with a previous laboratory study which showed "error" rates up to 26% in some devices [1]. The difference between turbine and Wright meter was small but the difference between Wright Meter and Fleisch meter could be sufficiently large to lead to a change in a patient's treatment if the two readings were to be used inter-changeably. Most of the differences can be explained by the use of different scales to measure PEF. The Wright's meter that we used and the turbine spirometer give a PEF reading on the "old" Wright's scale that has been used since 1959 but the Fleisch pneumotachograph gives a more accurate PEF measurement [2,3]. Converting the Wright's PEF measurements to the new EU scale abolished most of the difference between the Wright meter and the Fleisch pneumotachograph with virtually identical measurements in the range of 80–350 L/min.

## Conclusion

In laboratory studies, the Wright's meter has been shown to over-estimate the PEF in the mid-range compared with pre-determined machine-generated airflows [2,6,8]. The scales of the Wright meters and Mini-Wright meters were changed to the new EU scale in September 2004 and the results of this experiment indicate that measurements from a Wright meter using the new EU scale are likely to be very similar to those from a Fleisch pneumotachograph but only if the same technique is used on both devices. It is also important to note that patients and doctors should not compare readings made on different Wright meters (new scale or old scale) when deciding on whether to change a patient's treatment. The key point is that a patient's serial PEF should be measured on a single type of device using a consistent technique and measurements made on different machines should not be used to monitor a patient's progress.

## Competing interests

The author(s) declare that they have no competing interests.

## Authors' contributions

TB designed the study, collected the data and drafted the manuscript

RBOD designed the study, performed the statistical analysis and drafted the manuscript.

## Pre-publication history

The pre-publication history for this paper can be accessed here:



## Figures and Tables

**Table 1:** Patient characteristics

Patient Characteristics	
Number	36
Male/Female	18/18
Mean Age (sd)	64 (12.8)
Mean PEF on standard Wright Meter (SD and range)	308 (SD 137, range 80 to 570)
Mean PEF as percent predicted	75%
Diagnosis	12 Asthma 9 COPD 8 Breathlessness, not yet diagnosed 3 Lung Cancer 1 Aspergilloma 1 Bronchiectasis 1 Byssinosis 1 Idiopathic Pulmonary Fibrosis

**Table 2:** Mean PEF recorded using both techniques on three devices.

Technique	Mean PEF (l/min) (SD)	PEF as % predicted	Variance from Wright's PEF And t-test for difference
Wright Meter PEF technique	308 (137)	75% predicted	Not applicable
Wright Meter FVC technique	284 (131)	69% predicted	7.8% lower p < 0.0002
Turbine Spirometer PEF technique	291 (138)	71% predicted	5.5% lower p < 0.0002
Turbine Spirometer FVC technique	258 (128)	63% predicted	16.2% lower p < 0.0001
Fleisch Spirometer PEF technique	248 (118)	60% predicted	19.5% lower p < 0.0001
Fleisch Spirometer FVC technique	234 (113)	57% predicted	24.0% lower p < 0.0001
Wright Meter PEF converted to new EU PEF scale	266 (130)	65% predicted	13.6% lower
